# Physics-informed deep learning framework to model intense precipitation events at super resolution

**DOI:** 10.1186/s40562-023-00272-z

**Published:** 2023-04-18

**Authors:** B. Teufel, F. Carmo, L. Sushama, L. Sun, M. N. Khaliq, S. Bélair, A. Shamseldin, D. Nagesh Kumar, J. Vaze

**Affiliations:** 1 Department of Civil Engineering, Trottier Institute for Sustainability in Engineering and Design, McGill University, Montreal, Canada; 2 Ocean, Coastal and River Engineering (OCRE) Research Centre, National Research Council Canada, Ottawa, Canada; 3 Meteorological Research Division, Science and Technology Branch, Environment and Climate Change Canada, Dorval, Canada; 4 Department of Civil and Environmental Engineering, University of Auckland, Auckland, New Zealand; 5 Department of Civil Engineering, Indian Institute of Science, Bangalore, India; 6 CSIRO Land and Water, Canberra, Australia

**Keywords:** Deep learning, Regional climate model, Convection permitting model, Intense precipitation, Engineering scale

## Abstract

Physical modeling of precipitation at fine (sub-kilometer) spatial scales is computationally very expensive. This study develops a highly efficient framework for this task by coupling deep learning (DL) and physical modeling. This framework is developed and tested using regional climate simulations performed over a domain covering Montreal and adjoining regions, for the summers of 2015–2020, at 2.5 km and 250 m resolutions. The DL framework uses a recurrent approach and considers atmospheric physical processes, such as advection, to generate high-resolution information from low-resolution data, which enables it to recreate fine details and produce temporally consistent fields. The DL framework generates realistic high-resolution precipitation estimates, including intense short-duration precipitation events, which allows it to be applied in engineering problems, such as evaluating the climate resiliency of urban storm drainage systems. The results portray the value of the proposed DL framework, which can be extended to other resolutions, periods, and regions.

## Introduction

Intense short-duration precipitation events can lead to catastrophic flash flooding in urban regions and their intensity/duration/frequency characteristics are thus widely used in the design of engineering systems, such as storm water drainage. In a warming climate, the intensity and frequency of extreme precipitation are expected to increase due to the increasing water-holding capacity of the atmosphere (e.g., IPCC 2013), which is increasingly being accounted for by practitioners during the design of civil infrastructure. Understanding projected changes to short-duration precipitation extremes is particularly important, given that rapid urbanization is occurring, leading to over two-thirds of the global population being projected to live in urban regions by 2050 (UN 2018). Urban regions influence regional weather and climate, as they are replete with anthropogenic heat and aerosol sources, store little water and obstruct atmospheric motion (e.g., Oke 1982; Huszar et al. 2014; Daniel et al. 2019). The urban heat island (UHI), characterized by higher temperatures in urban regions than surrounding areas, may contribute to strengthening convection which can lead to enhanced precipitation extremes (e.g., Shepherd and Burian 2003; Mölders and Olson 2004).

Regional climate models (RCMs) are useful tools to study projected changes to climate, which are being increasingly used to inform effective adaptation measures to cope with the impacts of global warming in many fields. However, short-duration precipitation extremes are generally associated with small-scale processes, such as deep convection, which are not resolved at the scales of most existing RCM simulations and are thus approximated through various parameterizations. The recent studies have shown that high resolution (i.e., convection permitting) RCM simulations can realistically capture short-duration precipitation extremes due to better representation of mesoscale dynamics, cloud microphysics, surface heterogeneity and orographic effects (Prein et al. 2015; Kendon et al. 2017; Diro and Sushama 2019; Teufel and Sushama 2022).

Development of effective adaptation and mitigation strategies requires information of intense precipitation changes at engineering/super scales (i.e., < 1 km spatial resolution). The previous studies have shown that climate simulations with advanced representation of urban regions in RCMs are able to adequately capture urban−climate feedbacks (e.g., Teufel et al. 2021). However, despite significant developments in computing technology, parallel programming architectures and improved representation of physical processes in climate models, high computational cost continues to be a major barrier in undertaking climate simulations at engineering scales for sufficiently longer periods. For example, a climate simulation at 250 m requires about 1000 times the computing resources required at 2.5 km over the same domain. In addition, ensembles of climate simulations are generally required to quantify uncertainty in climate projections and that further amplifies the required resources. The recent advances in machine learning (Brenowitz and Bretherton 2018; Reichstein et al. 2019) and its applications in various fields (e.g., Chung and Shin 2018; Ding et al. 2020; Pradhan et al. 2020; Stengel et al. 2020; Van et al. 2020; Ray and Chattopadhyay 2021; Barrera-Animas et al. 2022; Girihagama et al. 2022) provide an opportunity for developing hybrid approaches, combining physical understanding of atmospheric processes with machine learning architectures, to overcome this obstacle and advance studies on climate−urban infrastructure interactions (Wu et al. 2021) and informing design methodologies. In the recent years, deep learning-based image super-resolution (SR) models built using convolutional neural networks (CNNs) have been developed (e.g., Wang et al. 2015; Dong et al. 2016; Lai et al. 2017; Zhang et al. 2018) and applied to produce high-resolution physical fields in various domains (Li et al. 2009; Trinh et al. 2014; Vandal et al. 2017; Xie et al. 2018; Stengel et al. 2020). The CNN-based SR approach is data-driven and does not require solving complex analytical formulations, which dramatically decreases the computational cost of generating high-resolution data once the parameters of CNNs are properly trained and validated.

This study focuses on the development, validation and application of a novel framework combining the physically based regional climate model GEM (Global Environmental Multiscale) outputs with deep learning techniques to efficiently generate precipitation information at applied engineering-oriented fine spatial scales. Such fine resolution information is extremely useful for evaluating climate resiliency of urban infrastructure systems to flash flooding in urban centers such as Montreal, where flash flooding caused by high-intensity rainfall is a recurrent problem. Montreal is the second largest city of Canada and is the major economic hub in eastern Canada. Weather related disruptions can trigger cascading impacts on various interconnected urban systems in Montreal. Given such a high impact of short-duration precipitation extremes in urban regions, the objective of this study is to develop an efficient and physically consistent emulator for intense precipitation during the summer season for Montreal, at the sub-hourly time scales associated with deep convection, to support studies on climate resilience of various urban systems. In addition, the goal is to keep the development as generic as possible so that the developed framework can be extended to other seasons, periods and regions of the world.

The remainder of this manuscript is organized as follows: “Methodology” section describes the machine learning framework, the physical climate model, and the experiments performed. “Results” section presents the outputs obtained from the machine learning model and compares them to the climate model outputs. Finally, “Discussion and conclusions” section provides discussion, conclusions and future avenues of research. Main highlights of the study are also discussed in this section.

## Methodology

### Deep learning framework

Given that precipitation evolves in both space and time, the objective of generating high-resolution (HR) precipitation from low-resolution (LR) precipitation is functionally similar to the problem of video super-resolution (VSR). VSR differs from single image SR in that temporal relationships are exploited by combining the information from multiple LR frames to reach better quality results. Many existing VSR methods approach the problem by combining a batch of LR frames to estimate a single HR frame, effectively dividing the task of VSR into a large number of separate multiframe SR subtasks (Caballero et al. 2017; Liu et al. 2017; Makansi et al. 2017; Tao et al. 2017). However, generating each output frame separately reduces the method’s ability to produce temporally consistent HR frames, often resulting in artifacts.

**Fig. 1:**
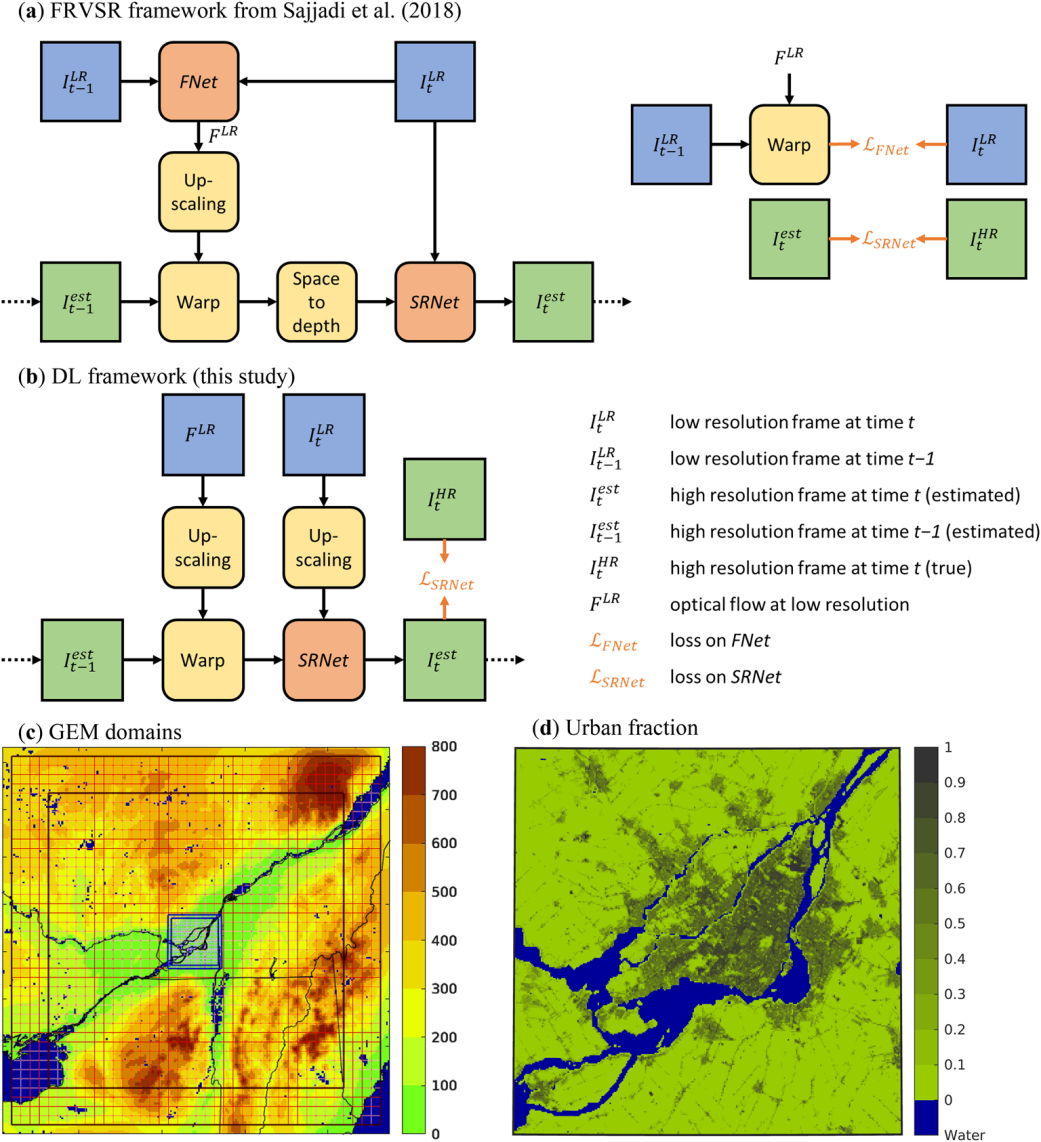
**a** The architecture of the FRVSR framework by Sajjadi et al. (2018). Blue boxes are used for LR input data, yellow boxes for field operators, green boxes for HR data and red boxes for CNNs. Loss terms are shown in orange. **b** Proposed deep learning framework for precipitation. **c** GEM computational domain at 2.5 km (brown; every 5th grid cell shown) and 250 m (blue; every 20th grid cell shown). The outer thick lines represent the model domain, while the inner thick lines represent the model free domain. Background colors represent orography (m asl). **d** Urban fraction in the 250 m domain. Water bodies are shown in dark blue

The deep learning (DL) framework used in this study is inspired by the one proposed by Sajjadi et al. (2018), named frame-recurrent video super-resolution (FRVSR; Fig. 1a). This recurrent approach passes the previously estimated HR frame as an input for the following iteration. Information from past frames can thus be propagated to later frames which helps the model to recreate fine details and produce temporally consistent videos, significantly outperforming other state of the art methods.

In this study, two physical considerations are taken into account to adapt the FRVSR approach to the task of generating SR precipitation. First, in VSR the displacements from one frame to the next (called optical flow) are typically estimated from the frames themselves—Sajjadi et al. (2018) used an encoder−decoder CNN for this task. The same approach could be chosen for SR precipitation, but instead, the physical fact that precipitation is advected by wind is used to derive optical flow, which has the advantage that flow estimates are available at all times and locations, given that wind is a continuous field. This approach also avoids having to train an additional CNN and needing to merge two loss terms. Second, the CNNs in Sajjadi et al. (2018) operate in LR space, enabling faster training, but imposing some constraints on the choice of the upscale factor (4 × in their case). This is undesirable in SR precipitation, where the upscale factor may vary depending on the specific resolutions of the HR and LR climate simulations. In addition, applying transposed convolutions or similar methods to generate HR outputs can result in undesirable gradients and artifacts—for these reasons, the CNN in the proposed SR precipitation framework is set to operate in HR.

The estimates of FRVSR tend to improve with time, i.e., for the first frame of a scene, its performance cannot exceed that of a single-frame image reconstruction method. For subsequent frames, the framework is able to leverage past information, thus producing better estimates. One implication of this is that if movement between consecutive frames is too fast, each frame will appear relatively independent from the previous ones, and a significant fraction of the new frame will not have been previously seen by the DL model, which would degrade its performance. To avoid this, displacements between frames should be small—in the case of SR precipitation, this can be achieved by using the highest feasible temporal resolution, as constrained by the timestep of the climate model.

Figure 1b shows the DL framework used in this study. The inputs consist of LR precipitation fields and LR storm motion estimates (derived from cloud-level winds). LR information first needs to be upscaled to HR, which is accomplished using bilinear interpolation on precipitation and each storm motion component (*u*, *v*). The HR storm motion is then used to advance the previous HR precipitation estimate in time, which is then used alongside the upscaled LR precipitation as inputs to SRNet, which outputs the new best estimate of HR precipitation. The above procedure is then repeated for each subsequent timestep. SRNet is a 5-layer CNN in which the convolutional kernel of each layer is 3 × 3 and each layer outputs 16 feature maps. The rectified linear unit (ReLU) function is used as the activation function for all convolutional layers. The loss function is defined as the mean squared error (MSE) of the reconstruction (for all grid cells and all timesteps).

### Climate model and simulations

The limited area version of the physically based model GEM (Côté et al. 1998; Girard et al. 2014) is used to perform simulations for the summers (June–August) of 2015–2020, at 2.5 km (low) and 250 m (high) resolution. The experimental domains for the two resolutions are shown in Fig. 1c, with the smaller 250 m resolution domain covering the city of Montreal. The urban coverage for Montreal and surroundings is shown in Fig. 1d. GEM is used for numerical weather prediction at Environment and Climate Change Canada (ECCC). The land part of the model is represented using the Canadian Land Surface Scheme–CLASS (Verseghy 2011), while the urban regions are represented by the Town Energy Balance (TEB; Masson 2000) model. Condensation processes are computed by a double-moment microphysics scheme (Milbrandt and Yau 2005). More details on the parameterizations used can be found in Diro and Sushama (2019). The urban climate simulation at high resolution is driven at the lateral boundaries by the low resolution GEM simulation, which is in turn driven by ERA5 reanalysis data (Hersbach et al. 2020) from the European Centre for Medium-Range Weather Forecasts. GEM outputs at 1-min intervals from both simulations constitute the input data for the DL framework, and match the timestep of the LR simulation. The HR simulation uses a 10 s timestep, but the highest feasible temporal resolution is constrained by the coarser timestep of the LR simulation (60 s).

### Deep learning experiments

It is fundamental to note that the HR GEM simulation is driven by the LR GEM simulation only at the lateral boundaries, which means that the HR simulation has considerable freedom to develop its own precipitation evolution, which often differs from the LR simulation in precipitation coverage and intensity. Although expected, these differences make it unadvisable to train the DL framework using the HR and LR simulation outputs directly. Instead, a two step process is developed: first, the DL framework is trained using the HR data and LR data generated from it (this is the method by which VSR frameworks are usually trained); and second, the trained framework is applied to the LR simulation outputs to generate HR data.

For the first step, it is desirable to generate LR data in which precipitation follows the spatiotemporal evolution of the HR data as closely as possible, given that during training the DL framework will strive to minimize errors in both space and time in the reconstruction of HR precipitation. This generated LR data (LR_G_) is obtained by simply averaging the HR fields over each LR grid cell. Here, the data for the summers of 2015–2018 is used as the training dataset, and the data for 2019 is used as the validation dataset. Finally, the data for 2020 are used as the test dataset.

For the second step, the unseen LR simulations outputs for the entire 2015–2020 period are used as inputs to the trained DL framework. The HR outputs from the framework will closely follow the spatiotemporal evolution of the LR data, which differs from the HR truth. Given this, and the focus on high-intensity precipitation events, the performance of the framework needs to be assessed in terms of statistical similarity, such as the ability to reproduce the precipitation frequency–intensity relationships, often used for the design of urban drainage networks.

## Results

### Reconstruction of HR information from LR_G_ data

The performance of the DL framework is assessed by comparing its estimates to the HR truth, for unseen data in the testing period (i.e., summer of 2020). Comparisons are performed both for select intense precipitation events, and in terms of overall statistics for the testing period.

**Fig. 2:**
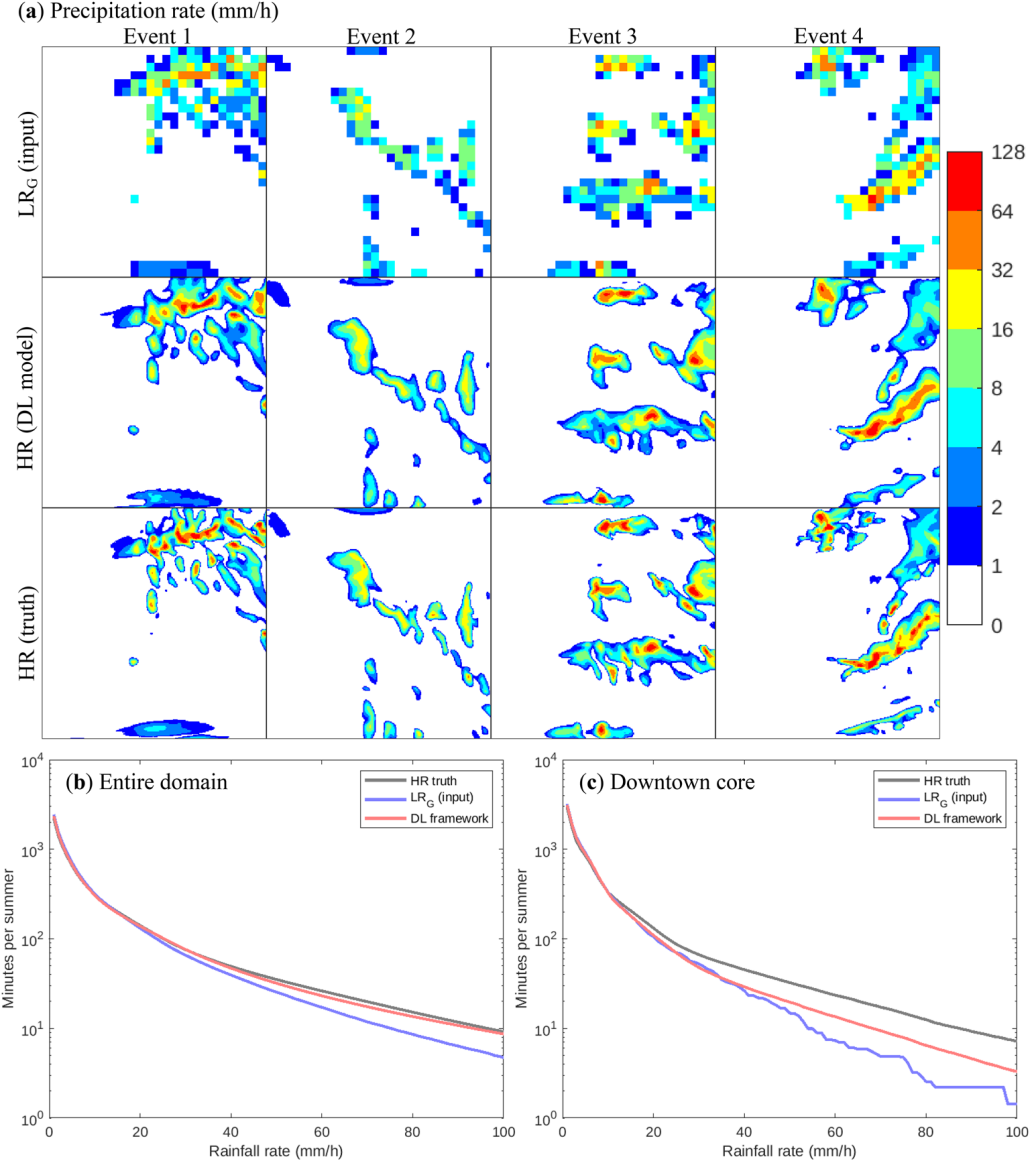
**a** Precipitation fields (mm/h) for select high-intensity precipitation events, from LR_G_ (top), DL model (middle) and HR truth (bottom). **b** Frequency–intensity relationships for the three datasets in **a**, over the entire HR domain. **c** As **b**, but for Montreal’s downtown core

Figure 2a shows the precipitation intensities for 1-min snapshots during selected heavy precipitation events. It can be seen that the DL framework performs very well compared to the HR truth, being able to reproduce many details not visible in the LR_G_ input for all of the events. For instance, the HR truth for event 1 shows the presence of many cores (local maxima) of heavy precipitation. The DL framework is able to realistically capture most of those cores, being only slightly smoother than the HR truth. The same is true for events 3 and 4, although for the latter, the finest structures (only few HR gridcells wide) are not fully reproduced. The DL framework also performs very well at delineating the regions with precipitation intensities above 1 mm/h and it is also able to emulate the spatial structure of precipitation cores, in which high-intensity precipitation is surrounded by lesser precipitation intensities, a feature not seen in LR_G_, where a grid cell with high-intensity precipitation can be adjacent to one with zero or negligible precipitation.

To further assess its performance, the ability of the DL framework to reproduce the precipitation intensity−frequency relationship is evaluated. In Fig. 2b, it can be seen that the frequency of very heavy precipitation (above 20 mm/h) is underestimated when averaging the HR data over a LR grid. For the heaviest intensities (100 mm/h and above), the frequency is underestimated by up to 50%. The DL framework is able to well reconstruct the frequency of heavy precipitation, reversing most of the underestimation present in the LR_G_ input, and being close to the HR truth over the entire range of precipitation intensities. Since the performance of the framework for specific locations within the domain is also of interest, Fig. 2c shows the intensity−frequency curves for Montreal’s downtown core. Compared with Fig. 2b, the underestimation of the heaviest precipitation rates is more pronounced in LR_G_, reaching close to 80%. The DL framework is able to correct a significant fraction of the underestimation, but not its entirety. One potential explanation would be higher frequency of precipitation events with very fine spatial structures over the downtown core, which are not fully reproduced by the DL framework (as previously mentioned).

### Generation of HR information from actual LR data

Given that the developed DL framework performs well at reconstructing the HR truth from the LR_G_ data (see “Reconstruction of HR information from LRG data” section), the next step is to apply the framework to the actual LR data and assess the realism of the generated output in terms of its similarity to the HR truth. Given that the fields cannot be directly compared at the grid cell level due to the differences between LR and HR GEM, measures of statistical similarity are used.

**Fig. 3:**
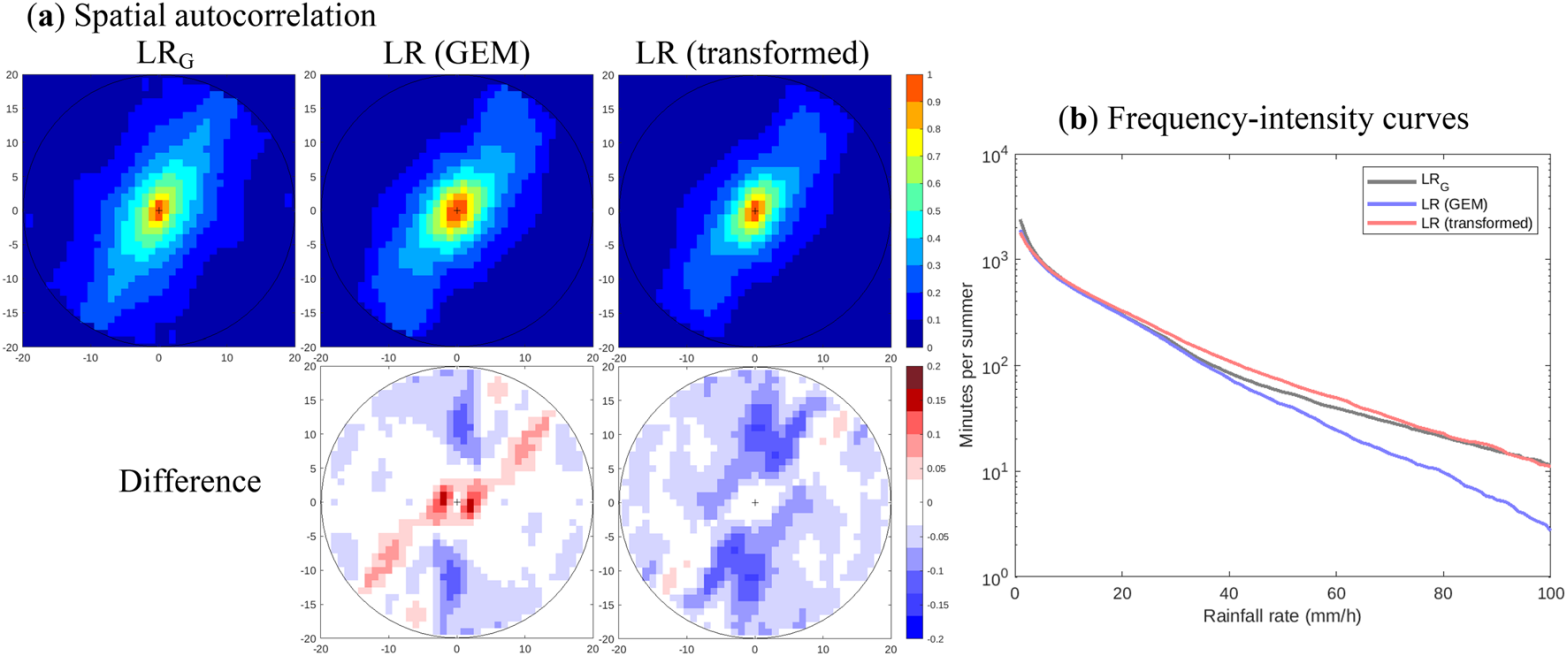
**a** Median spatial autocorrelations in LR_G_, LR (GEM) and LR (transformed), and their differences with respect to LR_G_. **b** Frequency–intensity relationships for the three datasets in **a**

GEM simulations at LR exhibit a significantly greater degree of spatial autocorrelation than the LR_G_ data created from HR fields (Fig. 3a). This is not surprising, considering that the actual resolution of a climate model is around 4–6 times coarser than its grid spacing, which means that the LR_G_ data are expected to have a greater amount of spatial detail (originally resolved at HR) than the corresponding LR data.

Given that the DL framework is trained using the LR_G_ data, it is desirable to transform the actual LR data so that it better resembles the LR_G_ data. The transformation applied reduces spatial autocorrelation (Fig. 3a) and is functionally similar to a sharpening filter. The transformation is performed by convolution with a 3 × 3 kernel, in which the center element has a value of 1.88, the four corners are zero, and the other four elements are − 0.22 (the sum of all elements equals one as to not introduce bias). These values are chosen with the goal of improving the frequency−intensity relationship of the LR data, which is much closer to LR_G_ after the transformation (Fig. 3b).

**Fig. 4:**
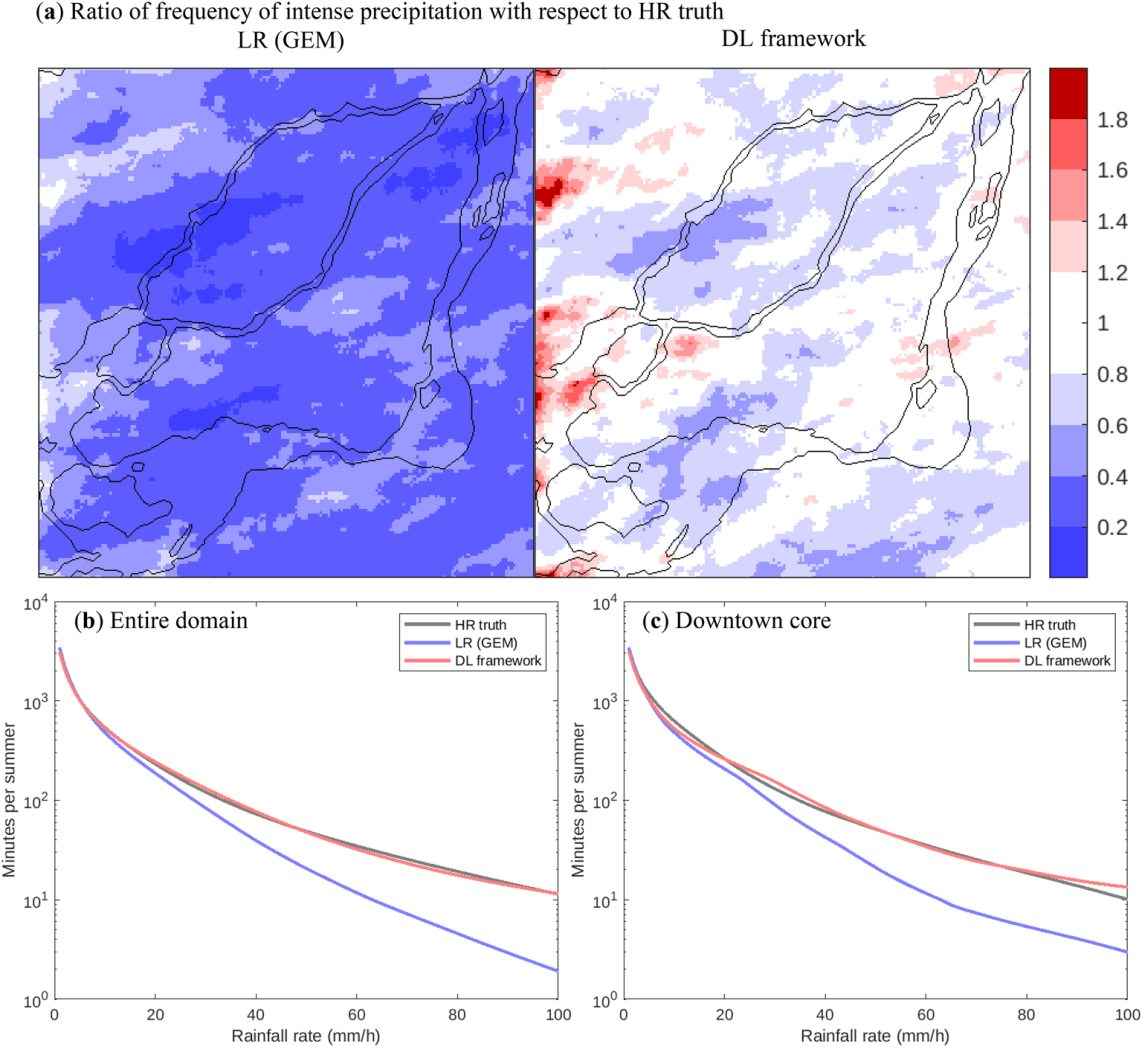
**a** Ratio of spatial frequency of rainfall intensity above 50 mm/h with respect to HR truth, from LR GEM (left) and DL model (right). **b** Frequency–intensity relationships for the three datasets in **a**, over the entire HR domain. **c** As **b**, but for Montreal’s downtown core

Applying the trained DL model to the transformed LR data produces HR estimates that closely follow the spatiotemporal evolution of precipitation in LR GEM, but with the statistical characteristics of HR GEM. Figure 4a shows that LR GEM significantly underestimates the frequency of occurrence of torrential precipitation (above 50 mm/h), which is greatly improved (i.e., much closer to the HR truth) after applying the DL framework. It is worth noting that the locations of high values in the DL outputs resemble those of LR GEM, which are not the same as in HR GEM. Given the relative rarity of such heavy precipitation, the differences in the location of the maxima are a consequence to the small sample size (6 years) and not indicative of long-term means. In terms of the precipitation intensity−frequency relationship (Fig. 4b), the DL framework produces results that are very close to the HR truth over the entire intensity range (from 1 to 100 mm/h). Even for specific areas, such as Montreal’s downtown core (Fig. 4c) the performance of the DL framework is quite adequate and much better than the original LR data.

## Discussion and conclusions

In this study, a physics-informed deep learning framework that enhances the resolution of precipitation from 2.5 km to 250 m (i.e., by a factor of 10) is developed and tested for Montreal, which is the second largest city in Canada, using outputs from a physically based regional climate model. It is noted that the deep learning framework is able to recreate fine details and produce temporally consistent precipitation fields by taking into account physical atmospheric processes, such as the advection of precipitation by wind, which is the novelty of this study. The results show that the deep learning model is capable of capturing many of the fundamental characteristics of intense short-duration precipitation events at fine spatial scales, such as their coverage, intensity and spatial structure.

When applying this framework to outputs from coarser climate simulations in future studies, it is important to account for the fact that the actual resolution of the precipitation data is coarser than its grid spacing, meaning that the data will be overly smooth and preprocessing (e.g., sharpening) would be required to increase the quality and realism of the estimates produced by the framework. Potential future improvements to this framework include taking into account the spatial structure of precipitation and/or controlling variables (e.g., buoyancy, wind speed/shear), and improvements to storm motion estimates, given that storms often influence atmospheric circulation at local scales and thus not always follow regional-scale winds. We intend to explore such ideas in future research.

Finally, the benefits of a properly trained deep learning framework capable of generating engineering scale precipitation information cannot be overstated. For a fraction of the computational cost of conventional methods that involve high-resolution numerical regional climate models, long simulations of precipitation with ample spatial and temporal detail can readily be performed, and even generating several ensembles of simulations is readily feasible, which is fundamental for quantifying uncertainty in climate projections. This advancement in modeling intense precipitation events using physics-informed deep learning framework will facilitate the development of effective adaptation and mitigation strategies for the climate challenges being faced by highly interconnected engineering systems in complex urban environments. It is hoped that the outcomes of this study and the developed theoretical framework will trigger several additional studies in other urban regions of the world.

## Data Availability

All data used in this study can be accessed at: https://zenodo.org/record/6631996
